# The Institutional Worker

**Published:** 1920-12-25

**Authors:** 


THE
### INSTITUTIONAL WORKER
**Being a Special Supplement to "The Hospital"**

### OUR BUREAU OF INFORMATION.

### Rules for Correspondents

1. Every letter must be accompanied by the coupon to be cut from the back cover (inside page) of The Hospital, current issue, and must contain the name and address of the correspondent with pseudonym for publication if desired. Replies by post cannot be given save under exceptional circumstances at the Editor's discretion.

2. Letters from Approved Homes in reply to special needs published in the Bureau should state terms and full particulars, and be sent prepaid under cover to the Editor of the Bureau with name written across coupon for identification.

3. Proprietors of Homes which have not yet been entered on the List of Approved Homes, but have spare accommodation likely to suit special needs, are invited to write for an application form for registration. The fee for registration, which includes two announcements of the Home in the Bureau and other privileges, is 10s.

4. All communications to be addressed to the Editor of The Hospital, 28 Southampton Street, Strand. London, W.C. 2, and marked "Bureau of Information."

### INSTITUTION FACTS AND FIGURES.

### A Nurses' Library.

#### Answer to November Question.

The question was:

Describe fully the most practical way of forming and running a nurses' library suitable for probationers, staff nurses, and sisters The description should include an outline of necessary rules, the administration, the selection of books, finance, and a method for keeping the library up to date and disposing of old books.

**"Wales" replies:**

In commencing a nurses' library, a meeting of the staff should be held and a committee formed consisting of at least six members, according to the size of the hospital. Sisters, nurses, and probationers should be represented. The members of the committee should be chosen by ballot; they should be well read and well informed on current topics. The next step should be to appoint a librarian and a treasurer.

The librarian should be responsible for the books, their issuing, numbering, and cataloguing. Valuable help can be had from the librarian of the town public library, who is usually very ready to give advice.

The treasurer should be responsible for the finance, and should keep the accounts on a business method; a balance sheet should be presented either yearly or half-yearly.

The committee should buy the books, helped by a list submitted by the librarian. A catalogue of the latest books can always be had from the publishers or an up-to-date bookshop; matron, or someone else with a broad outlook, to act as censor. Members should be allowed to choose or make suggestions at a meeting to be held, say, monthly; these should be made a note of by the librarian, and, if approved, such books bought with the next consignment. This helps to keep the library up to date, and gives the staff special interest in it.

Funds.

1. A donation from each member to start the library, then a monthly subscription of 3d. to 6d., according to numbers likely to join.

2. Sometimes it is possible to get a donation or grant from the Hospital Committee or some members of it towards the text-books for study, and in some hospitals they subscribe yearly a small sum to get up-to-date lecture books.

3. An American tea, or whist-drive, may be held, and past nurses and friends invited, in a large hospital. A good sum may be made in this way.

4. Nurses can ask their friends to hand over any books they have finished with. This is useful in the beginning to get a number, and any unsuitable can be weeded out. Books may often be purchased from the leading lending libraries at a reduced price.

Rules.

1. Books to be exchanged at stated times. Librarian to keep records.

2. Study books to be given out in rotation as asked for, and not to be kept more than one week.

3. Any book lost or damaged to be replaced by the member responsible.

4. No books to be taken into wards or lent to the patients.

5. One book only issued at a time.

6. Fines to be imposed if kept over time.

Old books can be sold to second-hand shops, or may be taken in exchange when buying new ones.

### Dental Radiography.

It was interesting and gratifying to note at the recent Medical Exhibition the advances made in apparatus for dental radiography. Until lately this branch of radiography has not received the attention its usefulness merits. As a means of locating the presence of septic foci at the root of the teeth it is of the utmost value to the physician, and the dental surgeon recognises its usefulness as an aid to clinical methods of diagnosis. There is no doubt that the want of convenient apparatus hindered progress in the work. Though the technique is simple enough when mastered, it is somewhat tedious if an easily adjusted tube-holder is not available. The apparatus exhibited by several well-known makers should sweep away all difficulties. A complete unit for this work can be purchased for a reasonable sum. A small high-tension transformer, with apparatus for Coolidge tubes put up in a small cabinet and a tube-holder fitted with a pointer and mounted on an arm attached to the cabinet, which can be slung in any direction, complete the outfit. The advantages of this provision will be appreciated by those who are occupied on work of this nature.

### Question for December.

### THE WORKING OF THE NATIONAL HEALTH INSURANCE ACTS.

**What benefits, if any, have hospitals derived from the National Health Insurance Acts? Have they been helpful in the management of the Almoners' or Enquiry Officers' Department, and if so how?**

**(A minimum payment of 10s. 6d. will be made for each published answer.)**

RULES.

The following rules must be observed:—

1. Contributions must be written on one side of the paper. Conciseness and terseness are desirable features. The MS. must bear the name and address of the sender and be accompanied by coupon to be cut from the back cover (inside page) of the current issue of The Hospital. A pseudonym must be chosen if the name is not to be published.

2. Contributions must be addressed to the Editor of The Hospital Institutional Worker Supplement, 28 & 29 Southampton Street, Strand, London, W.C. 2, should reach him before the end of the current month, and be marked in the left-hand corner "Facts and Figures."

### QUESTIONS INVITED.

In connection with our Question Box, we offer every month five shillings for the best question sent in for consideration. The questions may be on any institutional subject and concern any department, from the secretary's to the porter's, or the matron's to the domestic staff; the one essential is that they must be practical and deal with points that have a definite relation to hospital or institutional administration, upkeep, finance, or management. Points relating to artificers' work, housekeeping, the laundry, out-patients, and other practical matters will be welcome. It is hoped that thus, when difficult or doubtful points arise in the course of their work, institutional workers will be encouraged to put them in the form of a question and send them to the Editor, The Hospital Bureau of Information, 28 Southampton Street, Strand, W.C. 2, marked "Question Box."

The best questions will be published in due course and our readers will be given the opportunity of answering them. Thus all institutional workers may be able to co-operate with the view of helping the work and smoothing the difficulties of each other. In short, we want the Question Box of the *Institutional Worker* to be freely used by every worker habitually.

### ENQUIRIES AND ANSWERS.

*THE SICK AND IN NEED.*

### Convalescent Home for Woman near Nottingham.

The woman patient to whom you refer may possibly be admitted to the Nottingham and Notts Convalescent Homes for Women, Grosvenor Road, Skegness. Admission is upon recommendation and payment of 7s. 6d. weekly, or without recommendation 21s. The length of stay is usually about three weeks. The Homes are closed for six weeks at Christmas, but if you apply now you will possibly stand a better chance of procuring admission for the woman as soon as the Homes are reopened.—Newark.

### Aid for Distressed Gentlewoman.

We regret we are unable to suggest any means of help in the direction you require. Have you applied for help to the Distressed Gentlefolks' Aid Association? If not, we advise you to do so. The address is 75 Brook Green, London, W. 6. Send your application in writing to the Secretary of the Association.—Mrs. O.

*EMPLOYMENT AND TRAINING.*

### Hospitals for Children.

You might write to the matrons of the following institutions and inquire if they have a vacancy for a probationer-nurse: The Nottingham Children's Hospital, beds thirty-seven; Jenny Lind Infirmary for Children, Unthank Road, Norwich, beds sixty; Sheffield Children's Hospital, Western Bank, Sheffield, beds sixty; and Rosehill Hospital for Sick Children, Torquay.—P. W.

### Recognised Training School in Scotland.

The Dumfries and Galloway Royal Infirmary has 100 beds. Dundee Royal Infirmary is a much larger training-school, having 400 beds. You will find a full list of Scottish training-schools, with particulars of training of each, in "How to Become a Nurse," published by the Scientific Press, Ltd., 28 and 29 Southampton Street, Strand, W.C. 2, price 2s. 10d. post free.—Jean.

### Nursing in New Zealand.

If you intend taking up rural nursing in New Zealand you will certainly require a maternity certificate. You can obtain a six months' course in maternity nursing in one of the New Zealand St. Helens hospitals under the Public Health Department; the fee payable is £10. You can, if you hold a certificate of three years' training in a recognised British training-school, secure State Registration in New Zealand, the fee for which is £1.—Corbell.

*MISCELLANEOUS.*

### Polishing Cloths for Brass and Copper.

The "Redio" polishing cloths (redio green), made of a cloth impregnated with powerful metal-cleaning preparation for brasses, etc., are very useful and save a lot of trouble in the cleaning of taps and other metal fittings, and are much cleaner than ordinary metal polish. The price is 9d. for an ordinary-sized cloth, or 4½d. for special sample size. Special discounts are allowed to hospitals. The manufacturers are the Redio Co., Ltd., Warwick Road, Hampton Wick, Middlesex.—Labour Saving.

### Surgeons' Rubber Gloves.

Rubber gloves may be repaired in the same fashion as the inner tube of a bicycle tyre. Cut up an old glove into sizes suitable for the needful repairs; cleanse the glove to be repaired, and stick on the patch with rubber solution; pressure should then be applied until the patch is firmly adherent. Dry sterilisation should not present the difficulty you have experienced. There is a difference of opinion as to whether this or the wet method is the better; the experience of some suggests that gloves last longer if dry sterilised. You should dry and powder the gloves on both sides, leaving them right side out; place them in pairs, thumbs together, fold them and draw them inside each other. Then put a piece of gauze in the double end to allow the steam to penetrate. Wrap the gloves up in a piece of muslin, with a little powder tied up in a piece of gauze: put them in the drum and pack loosely together with a piece of wool between each pair. Place a piece of muslin on the top, and sterilise in the ordinary way.—M. S.

### The Uncertificated Masseuse.

There is no law which precludes an uncertificated person from practising massage. Massage ought not to be practised except under medical supervision, and you can scarcely hope for work if your standard of training is not vouched for by competent authority. We urge you to supplement your training and obtain the certificate of the Chartered Society of Massage and Medical Gymnastics (until lately known as the Incorporated Society of Trained Masseuses).—G. F. M.

### Home for Consumptive Girl.

We would suggest the undermentioned institutions as being suitable for and likely to accept the girl you refer to as a patient: Royal National Hospital for Consumption, Ventnor; admission by Governor's letter of recommendation and a payment of £1 per week. Mount Vernon Hospital for Consumption, Northwood, Middlesex; admission by Governor's letter and a weekly payment of 15s. Apply to the Secretary, 7 Fitzroy Square, W. 1.—H. B.

